# High-power microwave ablation with preablation transarterial chemoembolization enhances ablation zone stability in patients with liver malignancies

**DOI:** 10.1007/s11547-026-02213-0

**Published:** 2026-05-05

**Authors:** Thomas J. Vogl, Daniel M. Dahm, Leon V. Stein, Fabian Finkelmeier, Hamzah Adwan

**Affiliations:** 1 Clinic for Radiology and Nuclear Medicine, Johann Wolfgang Goethe University, University Hospital Frankfurt, Theodor-Stern-Kai 7, 60590 Frankfurt Am Main, Germany; 2 Department of Gastroenterology and Hepatology, Johann Wolfgang Goethe University, University Hospital Frankfurt, Theodor-Stern-Kai 7, 60590 Frankfurt Am Main, Germany

**Keywords:** Transarterial chemoembolization, Microwave ablation, Ablation zone stability, Hepatocellular carcinoma, Liver metastases

## Abstract

**Purpose:**

We aim to assess whether high-power microwave ablation (MWA) in combination with preablation transarterial chemoembolization (TACE) is associated with greater ablation zone stability in patients diagnosed with hepatocellular carcinoma (HCC) or liver metastases. The primary endpoint was the percentage change in ablation zone area from baseline to late follow-up (T1–T3).

**Methods:**

In this retrospective, non-randomized, single-center study, 45 patients with HCC and 32 patients with liver metastases were analyzed. All patients underwent computed tomography (CT)-guided percutaneous MWA following TACE.

Protocols were stratified into a low-power protocol group (initiated at 60 W and limited to ≤ 100 W) and a high-power protocol group (initiated at 80 W and escalated to ≥ 120–150 W).

MRI (magnetic resonance imaging) follow-up was performed at 24 h (T1), 3 months (T2) and 6 months (T3), with manual measurement of the ablation zone area at each time point.

**Results:**

In the HCC cohort, high-power MWA was associated with significantly greater ablation zone stability (%ΔT1–T3 = − 32.95 ± 5.82%) compared to low-power protocols (%ΔT1–T3 = − 49.72 ± 6.91%;* p* < 0.001; Cohen’s *d* = 1.12). In the metastases cohort, high-power MWA also outperformed the low-power group (− 28.4% vs. − 42.1%; *p* < 0.001, RBC = 0.87; with consistent effect sizes).

**Conclusion:**

This retrospective single-center cohort study provides exploratory imaging evidence that higher power settings are associated with greater ablation zone stability following TACE, warranting prospective validation.

## Introduction

Hepatocellular carcinoma (HCC) and secondary hepatic malignancies, primarily metastasizing from colorectal cancer, are major causes of cancer-related morbidity and mortality worldwide. HCC is the sixth most common cancer and the third leading cause of cancer-related death worldwide, with more than 900,000 new cases and 830,000 deaths annually. The incidence is expected to rise further with increasing prevalence of chronic liver disease and metabolic factors [1, 2]. Additionally, a substantial proportion of patients present with unresectable disease or are not suitable surgical candidates due to impaired liver function or comorbidities. Curative treatment options include liver resection and liver transplantation, though many patients are ineligible for surgical interventions due to poor hepatic reserve [3]. In this clinical context, minimally invasive locoregional therapies have gained increasing importance. For these patients, image-guided thermal ablation techniques, such as microwave ablation (MWA) and radiofrequency ablation (RFA) represent established minimally invasive treatment options. MWA induces thermal cytotoxicity through microwave-frequency electromagnetic fields, while RFA generates heat using alternating electric currents [4]. MWA has gained preference in nonsurgical candidates because it enables higher intratumoral temperatures and generates larger, more homogeneous ablation zones. In addition, it is less susceptible to perfusion-mediated heat-sink effects compared with RFA [5]. The Barcelona Clinic Liver Cancer staging system recommends thermal ablation as a first-line therapy for very early-stage HCC when transplantation is not feasible [6]. In recent years, increasing evidence has supported the combined use of transarterial chemoembolization (TACE) followed by thermal ablation, particularly for lesions approaching or exceeding the size limits of ablation alone [7]. Beyond cytoreduction, TACE induces arterial flow reduction within and around the target lesion, thereby mitigating heat-sink effects and potentially enhancing the efficacy and predictability of subsequent thermal ablation [8]. Several recent studies have demonstrated improved local tumor control and progression-free survival for combined approaches compared with monotherapy, without a relevant increase in procedure-related morbidity [7, 9].

Recent technological advances have enabled the clinical implementation of high-power MWA systems exceeding 100 W. Early clinical experience with these systems suggests that higher power delivery may shorten ablation times and generate larger ablation zones compared with earlier low-power systems [10]. However, data on the temporal stability and predictability of ablation zones following high-power MWA, particularly in combination with preablation TACE, remain limited and largely heterogeneous. Combination therapy with TACE followed by thermal ablation has been shown to improve local tumor control compared with TACE alone, as reported in multiple clinical series [9]. Originally designed for primary liver tumors, TACE is also applied to metastases from colorectal and breast cancer [11]. Several studies suggest that MWA can achieve outcomes comparable to resection, including similar 5-year survival rates [12, 13], and in unresectable liver metastases, thermal ablation in combination with TACE has been explored as a local treatment option [14]. Few studies, however, have systematically examined how MWA power settings influence ablation zone dynamics, particularly when combined with TACE. Emerging evidence suggests that outputs up to 150 W may improve completeness, shorten procedure time, and enlarge ablation zones [10].

Ablation zone stability, conceptualized as reduced contraction of the treated area over time on imaging, may represent an exploratory imaging marker of sustained necrosis, although it is not a validated surrogate endpoint. The purpose of this study was to investigate whether high-power protocol is associated with increased ablation zone stability following percutaneous MWA performed after TACE for patients with HCC or liver metastases.

## Material and methods

### Study design and ethics

This retrospective, single-center study included computed tomography (CT)-guided percutaneous MWA procedures performed at our institution between August 2017 and February 2024. The ethics committee approved this retrospective analysis (approval number: 2025–2435), which was conducted in accordance with the Declaration of Helsinki. Informed consent was waived due to the anonymized nature of the data and the retrospective study design.

### Patient selection

**Fig. 1:**
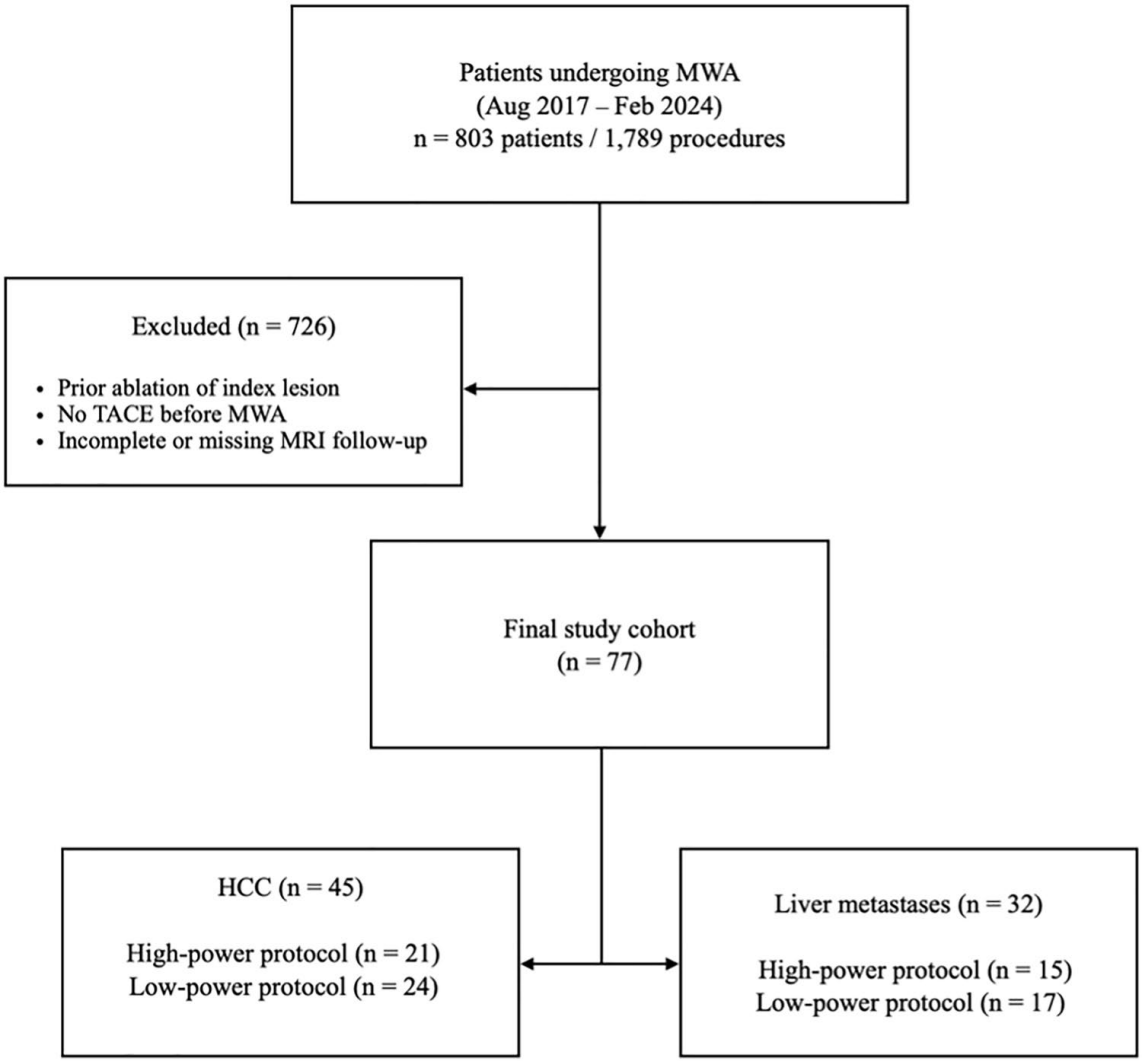
CONSORT-style flow diagram summarizing patient enrollment, exclusions, and allocation to high- and low-power MWA protocol groups

During the study period, a total of 803 patients underwent 1,789 MWA procedures at our institution. Patients were eligible if they had a confirmed diagnosis of HCC or liver metastases and underwent MWA following TACE. Furthermore, they required standardized contrast-enhanced magnetic resonance imaging (MRI) follow-up at three time points: within 24 h post-ablation (T1), 3 months (T2), and 6 months (T3), respectively. Exclusion criteria were prior index-lesion ablation, absence of TACE, or incomplete imaging follow-up. After applying these criteria, the final study cohort comprised 77 patients (HCC n = 45; hepatic metastases n = 32). (Figure 1).

### TACE and MWA procedures

TACE was performed using up to 10 ml/m^2^ of ethiodized oil (Lipiodol® Ultra-Fluid, Guerbet, Germany), with or without 180–225 mg of 50 µm degradable starch microspheres (DSM, EmboCept® S, PharmaCept GmbH, Germany). We applied Mitomycin C (8 mg/m^2^) and/or Cisplatin (20 mg/m^2^). The median interval between the last TACE and subsequent MWA was 22.5 days (interquartile range (IQR) 17–28) for low-power versus 20.0 days (IQR 16–24) in the high-power group among HCC patients. In the metastases cohort, corresponding intervals were 39 days (IQR 32–46) and 26 days (IQR 21–33), respectively. Baseline characteristics of HCC and metastases cohorts are summarized in Table 1.

**Table 1:** Baseline characteristics of HCC and metastases cohorts

	HCC Cohorts			Metastases Cohorts		
Characteristic	Low-Power	High-Power	*p*–value	Low-Power	High-Power	*p*–value
Number of Patients (n)	24	21	–	17	15	–
Male [n (%)]	23 (95.8%)	21 (100%)	1.000^a^ (φ = 0.11)	5 (29.4%)	6 (40.0%)	0.798^b^ (φ = 0.10)
Female [n (%)]	1 (4.2%)	0 (0%)	–	12 (70.6%)	9 (60.0%)	–
Age (years)	66.3 ± 8.5	69.6 ± 6.8	0.18^a^ (RBC = 0.26)	53.0 [46.0–60.0]	56.0 [49.5–62.5]	0.41^a^ (RBC = 0.16)
BMI (kg/m^2^)	27.2 ± 4.3	26.5 ± 3.9	0.520^a^ (RBC = − 0.11)	26.7 [25.0–28.3]	26.2 [24.7–28.4]	0.63^a^ (RBC = 0.13)
MELD Score	12.3 ± 3.0	11.8 ± 2.7	0.440^a^ (RBC = − 0.14)	–	–	–
Child–Pugh Class A [n]	12	11	0.710^b^ (φ = 0.05)	–	–	–
Child–Pugh Class B [n]	12	10	0.710^b^ (φ = 0.05)	–	–	–
Cirrhosis (%)	100%	100%	–	–	–	–
Tumor Area (cm^2^)	2.84 [2.00–3.79]	2.89 [2.00–3.79]	0.517^a^ (RBC = 0.11)	6.4 [5.3–7.2]	6.8 [5.6–7.5]	0.570^a^ (RBC = − 0.11)
BCLC Stage A (n)	14	11	0.710^b^ (φ = 0.05)	–	–	–
BCLC Stage B (n)	10	10	0.710^b^ (φ = 0.05)	–	–	–
Days since last TACE	22.5 [17.0–28.0]	20.0 [16–24.0]	0.570^a^ (RBC = 0.09)	39.0 [32.0–46]	26.0 [21.0–33.0]	0.508^a^ (RBC = 0.15)

Continuous variables are presented as mean ± standard deviation (SD) or median (IQR), as appropriate, and categorical variables as n (%). Summary statistics were selected according to distribution within each cohort. Effect sizes are reported as RBC (rank-biserial correlation) and φ (phi coefficient). All tests were two-sided. BCLC, Barcelona Clinic Liver Cancer stage; Model for End-Stage Liver Disease, MELD; BMI, body mass index. Superscripts denote statistical tests: ᵃMann–Whitney U test; ᵇChi-squared test(*χ*^2^ )test (or Fisher’s exact test when expected cell counts were < 5)

### Probe selection and power stratification

**Fig. 2:**
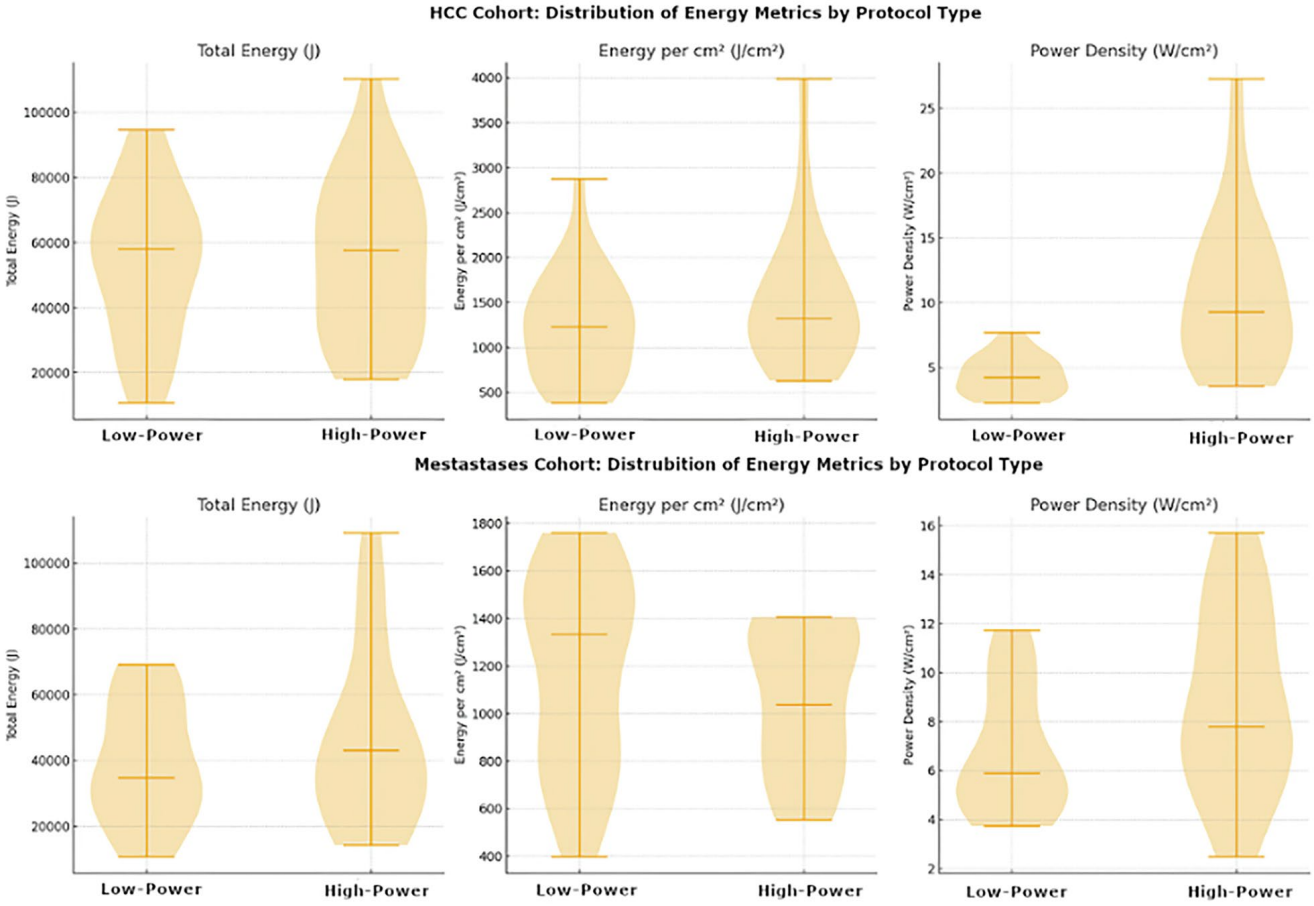
Distributions of delivered energy metrics by protocol type. Violin plots illustrate per-patient distributions of total energy (J), energy per cm^2^ (J/cm^2^), and power density (W/cm^2^) in the HCC and the metastases cohorts. While total energy and energy per cm^2^ overlapped between low- and high-power protocols, power density was significantly higher in the HCC high-power group (*p* < 0.001) and showed an upward trend in the metastases group (*p* = 0.089)

Various probes were used according to lesion size, location, and operator preference, including Solero (14 and 20 cm; AngioDynamics, Latham, NY, USA), Amica (HS Hospital Service, Aprilia, Italy), and Emprint^TM^ antennas (including CA15L2 and CA20L2; Medtronic, Dublin, Ireland). All systems were capable of delivering high-power output and were operated within manufacturer-recommended settings. Patients in each cohort were stratified into two groups based on the applied microwave power protocol. In both groups, ablation was gradually started. The low-power protocol group comprised patients whose ablations were initiated at 60 W and were not escalated beyond a maximum of 100 W. In contrast, the high-power protocol group comprised patients whose ablations were initiated at 80 W and were consistently escalated to at least 120 W (up to 150 W). No patient in the high-power group was treated exclusively below 120 W, ensuring clear technical separation between protocols. To characterize and verify technical separation between protocols, delivered energy parameters (time, total energy (J), energy per cm^2^ (J/cm^2^), power density (W/cm^2^) were analyzed (Fig. 2, Table 2). Power density (W/cm^2^) was calculated as input power divided by the active antenna surface area. This area-normalized metric differs from absorbed power density or specific absorption rate  (W/kg), which is typically reported in modeling studies, and was used pragmatically to compare protocol intensity.

**Table 2:** Delivered energy and procedural metrics by protocol type in the HCC and the metastases cohorts

Cohort	Metric	High-Power (mean ± SD/median)	Low-Power (mean ± SD/median)	Test	*p*–value	Effect size
HCC	Total Energy (J)	55,171 ± 25,156/57,600	53,492 ± 23,013/58,200	*t*-test	0.82	Cohen’s *d* = 0.07
	Energy/cm^2^ (J/cm^2^)	1,534 ± 510/1,322	1,244 ± 498/1,229	Mann–Whitney	0.19	RBC = 0.21
	Power Density (W/cm^2^)	10.8 ± 6.1/9.3	4.4 ± 1.5/4.2	Mann–Whitney	< 0.001	RBC = 0.87 (95% CI: 0.71–0.94)
	Total ablation time (min)	7.71 ± 3.27/8.0	9.22 ± 3.57/9.5	Mann–Whitney	0.168	RBC = − 0.24
Metastases	Total Energy (J)	45,820 ± 27,180/43,200	39,447 ± 18,753/34,800	Mann–Whitney	0.76	RBC = 0.08
	Energy/cm^2^ (J/cm^2^)	1,057 ± 490/1,037	1,184 ± 520/1,333	Mann–Whitney	0.21	RBC = − 0.22
	Power Density (W/cm^2^)	8.9 ± 3.9/7.8	6.8 ± 2.6/5.9	Mann–Whitney	0.089	RBC = 0.41 (95% CI: − 0.03–0.7)
	Total ablation time (min)	6.71 ± 3.44/7.0	7.27 ± 3.05/6.0	Mann–Whitney	0.495	RBC = − 0.15

Values are presented as mean ± SD and median [IQR], as appropriate. Statistical tests were selected based on Shapiro–Wilk normality results (independent *t*-test or Mann–Whitney U test, as appropriate). Effect sizes are reported as Cohen’s *d* for normally distributed variables or RBC with 95% confidence intervals

### MRI acquisition and image analysis

MRI follow-up was performed using standardized protocols to ensure consistency, including non-contrast T1- and T2-weighted coronal and transverse sequences (6 mm slice thickness), dynamic contrast-enhanced, and post-contrast T1-weighted imaging. Interobserver agreement was not assessed, as all measurements were performed by a single reader using a standardized and predefined measurement approach across all cases and time points. While this ensured internal consistency, formal quantification of measurement reproducibility (e.g., intraclass correlation coefficient or Cohen’s kappa) was not possible and should be addressed in future prospective studies. Contrast dosing was weight-adjusted, and scans were performed on 1.5-T and 3-T MRI systems (Siemens Healthineers).

Technical success was defined according to Ahmed et al. [15].

Tumor and ablation largest cross-sectional diameter (length and width, in cm) were manually measured by a single observer using consistent anatomical landmarks at three time points (T1, T2, T3). MRI was performed at three fixed time points: within 24 h (T1) to confirm technical success, at 3 months (T2) for early oncologic assessment, and at 6 months (T3). T2 served as a routine safety and efficacy control. The primary endpoint of this study was the percentage change in ablation zone area between T1 and T3, as this best reflects contraction dynamics.

The observer was blinded to MWA protocol to minimize bias. The cross-sectional tumor as well as the ablation area (cm^2^) were calculated at each time point using the following formula:

$$Area \, = \, length \, * \, width \, * \, (\pi /4)$$

Ablation zone stability was defined as the percentage change in area from T1 to T3, with smaller reduction suggesting greater effect.

### Statistical analysis

Statistical analyses were performed using JASP software (version 0.19.3, JASP Team, 2025). Normality of continuous variables was tested with the Shapiro–Wilk test. Depending on their distribution, groups were compared with independent *t*-tests or Mann–Whitney U tests. Effect sizes were reported as Cohen’s *d* for normally distributed variables and rank-biserial correlation (RBC) for non-parametric variables. Exploratory correlations, particularly between total energy delivery and ablation zone contraction, were assessed using bootstrapped Spearman’s ρ (1,000 replicates).

Independent predictors of ablation zone stability were identified using uni- and multivariate linear regression with 5,000 bootstrap replicates to obtain robust 95% confidence intervals (CI).

Survival outcomes were analyzed using Kaplan–Meier curves and compared using log-rank tests. Cox regression was applied to assess the influence of power protocol on survival, adjusting for tumor area, ablation margin, and total energy. All statistical tests were two-sided, and statistical significance was defined as *p* < 0.05. Given the retrospective nature of the study, no a priori sample size calculation was performed.

Box plots, violin plots, and the correlation plot were generated from original data using code refined with assistance from ChatGPT (OpenAI; GPT-5). All figures were verified by the authors.

## Results

### Baseline characteristics

A total of 45 patients with HCC were included (mean age 67.8 ± 7.8 years; 95% CI: 65.4–70.2), with 21 high-power (69.6 ± 6.8; 95% CI: 66.6–72.6) and 24 low-power MWA (66.3 ± 8.5 years; 95% CI: 62.9–69.7). Due to small sample sizes and potential deviations from normality (age: *p* = 0.022/0.048; body mass index (BMI): *p* = 0.035/0.041; Model for End-Stage Liver Disease (MELD): *p* = 0.027/0.033), group comparisons were performed using Mann–Whitney U tests. No significant differences were found in age (U = 186.5, z = − 1.34, *p* = 0.180, RBC = 0.16), BMI (27.2 ± 4.3 vs. 26.5 ± 3.9; U = 223.5, z = − 0.64, *p* = 0.521, RBC = − 0.11), or MELD (12.3 ± 3.0 vs. 11.8 ± 2.7; U = 217.5, z = − 0.77, *p* = 0.442, RBC = − 0.14). All HCC patients had cirrhosis, with comparable Child–Pugh (A/B: 12/12 vs. 11/10; Chi-squared test (*χ*^2^)(1) = 0.134, *p* = 0.714, phi coefficient  (φ) = 0.05). TACE–MWA intervals did not differ (*p* > 0.05).

In the metastases cohort (n = 32; median age 56.0 years, IQR 49.5–62.5), 15 received high-power (median 56.0 years, IQR 49.5–62.5) and 17 low-power MWA (median 53.0 years, IQR 46.0–60.0). No significant group differences were observed in age (U = 107.0, z = − 0.82, *p* = 0.412, RBC = 0.16), tumor area (median 6.4 cm^2^, IQR 5.3–7.2 vs. 6.8 cm^2^, IQR 5.6–7.5; U = 113.5, z = − 0.57, *p* = 0.569, RBC = − 0.11), or BMI (median 26.7 kg/m^2^, IQR 25.0–28.3 vs. 26.2 kg/m^2^, IQR 24.7–28.4; U = 111.0, z = − 0.49, *p* = 0.626, RBC = − 0.13). All baseline variables showed deviations from normality in Shapiro–Wilk testing (*p* < 0.05). Detailed characteristics are provided in Table 1.

### Technical success

Technical success was achieved in all patients, with complete tumor coverage at T1 (24 h). No patient required re-ablation.

### Ablation zone contraction

In the HCC cohort, the mean ablation zone contraction from T1 to T3 was significantly smaller in the high-power protocol group (− 32.95% ± 5.82%; 95% CI: − 35.44 to − 30.46; n = 21) than in the low-power protocol group (− 49.72% ± 6.91%; 95% CI: − 52.48 to − 46.96; n = 24), indicating greater ablation zone stability with the high-power protocol.

**Fig. 3:**
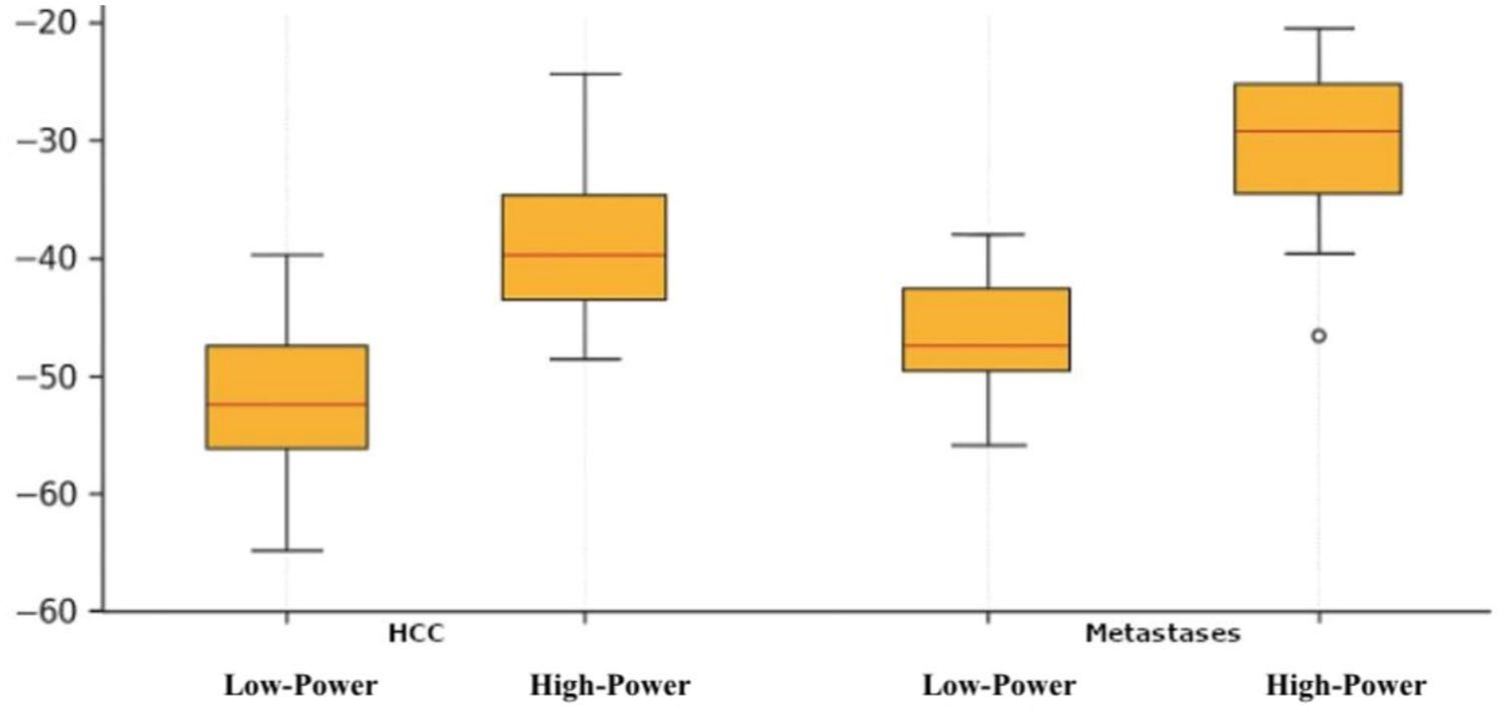
% Δ Ablation Zone Area (T1–T3) by group. High-power ablation protocols significantly reduced contraction in both the HCC and the metastases cohorts, indicating greater ablation zone stability

Both groups showed normal distribution (Shapiro–Wilk *p* > 0.05). The independent t-test confirmed a statistically significant difference (t(43) = 4.86, *p* < 0.001), with a mean difference of 16.77 percentage points (95% CI: 10.0–23.6). Effect size was large, with Cohen’s *d* = 1.12 (95% CI: 0.59–1.63). Results were validated with bootstrapped 95% CIs (percentile method, 5,000 replicates) to ensure robustness. This finding is illustrated in Fig. 3.

**Fig. 4:**
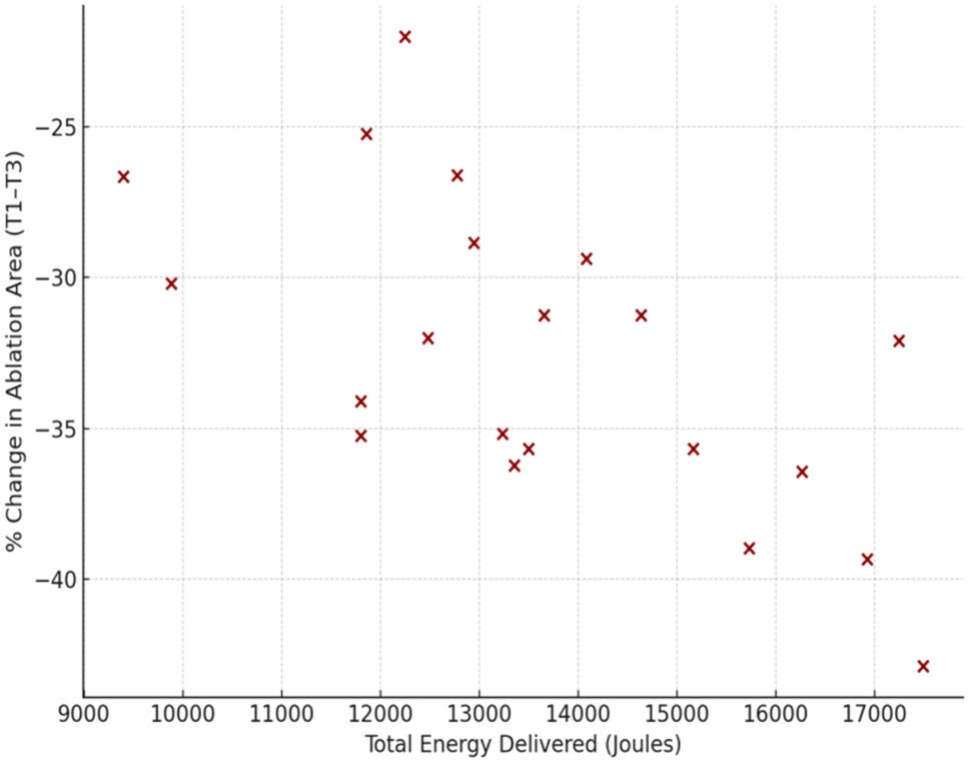
Correlation of Total Energy Delivered with %Δ Ablation Area (HCC High-Power). Higher energy delivery correlated with less ablation zone contraction (Spearman’s ρ = –0.48, *p* = 0.03), suggesting improved stability with increased energy

A moderate negative correlation was found between total energy delivered and contraction in the high-power group (Spearman’s ρ = − 0.48, *p* = 0.030; bootstrapped 95% CI: − 0.68 to − 0.21, 1,000 replicates; Fig. 4).

In the metastases cohort, the mean ablation zone contraction from T1 to T3 was − 28.4% ± 6.31% (95% CI: − 31.6 to − 25.2) in the high-power group and − 42.1% ± 7.81% (95% CI: − 45.8 to − 38.4) in the low-power group. Shapiro–Wilk indicated non-normal distributions (*p* = 0.031 and 0.017), justifying the use of the Mann–Whitney U test, which confirmed statistical significance (U = 34.5, z = − 3.56, *p* < 0.001). The effect size was very strong (RBC = 0.87; bootstrapped 95% CI: 0.72–0.94; 1,000 replicates; Fig. 3).

**Fig. 5:**
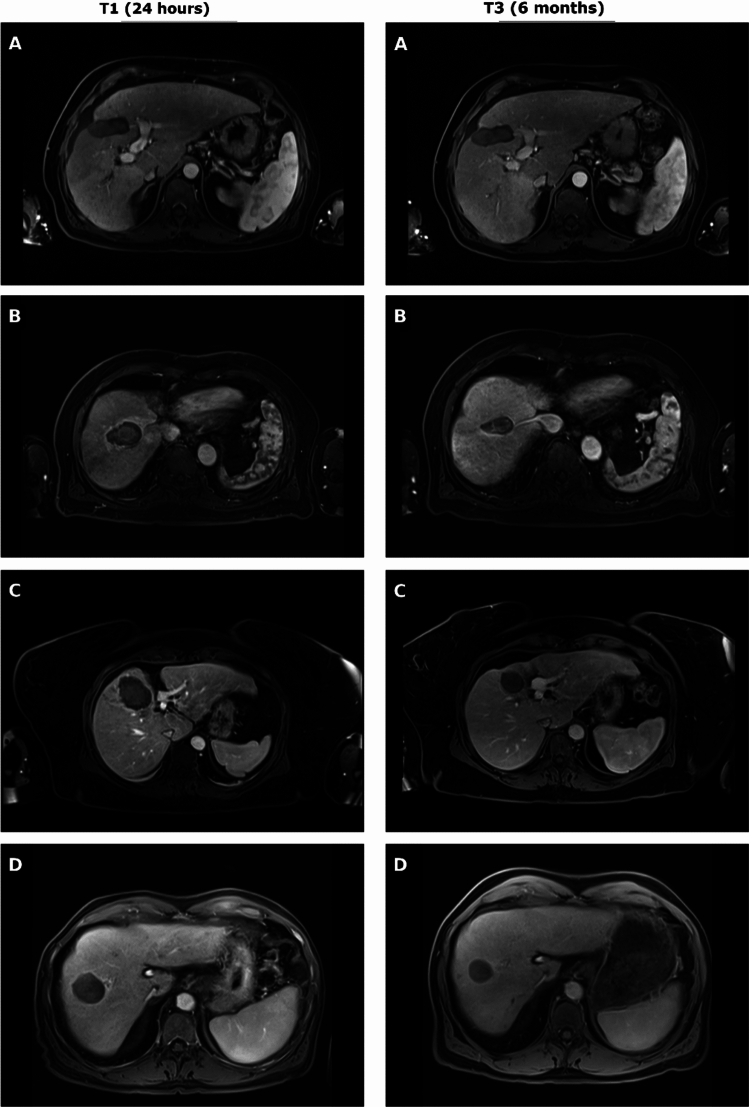
Representative MRI cases after TACE + MWA. **A** Case A, male, 77 years, HCC, high-power protocol: T1 shows complete coverage; at T3 contraction is moderate with preserved margins. **B** Case B, male, 66 years, HCC, high-power protocol: complete necrosis at T1; mild shrinkage at T3 with stable margins. **C** Case C, female, 79 years, breast cancer liver metastasis, low-power protocol: sharply demarcated ablation zone at T1; pronounced contraction by T3 with narrowing of the rim. **D** Case D, male, 68 years, HCC, high-power protocol: broad ablation zone at T1; limited contraction at T3 with margin integrity preserved

Representative case examples are presented in Fig. 5. In high-power cases (Cases A, B, and D), the ablation zones remained largely stable between immediate post-ablation imaging (T1, 24 h) and mid-term follow-up (T3, 6 months), showing only limited contraction while preserving safety margins. By contrast, in a patient with breast cancer liver metastasis treated with the low-power protocol (Case C), marked contraction reduced the ablative margin.

### Regression analyses

In the HCC cohort, univariate regression confirmed protocol type as a significant predictor of ablation zone contraction ( regression coefficient (β) = − 16.4, SE = 2.22, *p* < 0.001; 95% CI: − 20.9 to − 11.8; coefficient of determination (R^2^ ) = 0.59). Tumor size was also significant (β = 1.73, standard error (SE) = 0.34, *p* < 0.001; 95% CI: 1.05–2.40; R^2^ = 0.32). In multivariable analysis adjusting for energy, tumor size, MELD, and BMI, only protocol type remained significant (β = − 16.7, SE = 2.85, *p* < 0.001; 95% CI: − 22.6 to − 10.9; adjusted R^2^ = 0.63). MELD (β = − 0.07, *p* = 0.85), BMI (β = − 0.16, *p* = 0.77), and energy (β = 0.000098, *p* = 0.96) were not predictive. Collinearity was low (variance inflation factors (VIFs): protocol = 1.50, tumor size = 1.27, MELD = 1.74, BMI = 1.08, energy = 1.30).

In the metastases cohort, univariate regression identified protocol type as a significant predictor (β = − 13.6, SE = 4.42, *p* = 0.004; 95% CI: − 21.3 to − 6.2; R^2^ = 0.51). Tumor size showed a nonsignificant trend (β = + 0.98, SE = 0.56, *p* = 0.08; 95% CI: − 0.12–2.08; R^2^ = 0.09). In multivariable analysis adjusting for energy, tumor size, and BMI, only protocol type remained significant (β = − 12.8, SE = 4.89, *p* = 0.007; 95% CI: − 22.4 to − 3.2; adjusted R^2^ = 0.56). BMI (β = − 0.17, *p* = 0.62) and energy (β = − 0.000019, *p* = 0.78) were non-significant. Collinearity diagnostics showed no issues (VIFs: protocol = 1.12, tumor size = 2.38, BMI = 2.36, energy = 1.46).

Univariate and multivariate results are summarized in Table 3.

**Table 3:** Univariate and multivariate regression analyses for predictors of ablation zone contraction (%Δ T1–T3) in HCC and metastases cohorts

Cohort	Model	Predictor	β (SE)	95% CI	*p*–value	R^2^/Adjusted R^2^	Collinearity (VIF)
HCC (n = 45)	Univariate	Protocol (high vs. low)	− 16.4 (2.22)	− 20.9 to − 11.8	< 0.001	R^2^ = 0.59	1.00
		Tumor size (cm^2^)	1.73 (0.34)	1.05–2.40	< 0.001	R^2^ = 0.32	2.24
		MELD score	− 0.07 (0.37)	− 0.83–0.69	0.85	R^2^ ≈ 0.01	1.74
		BMI (kg/m^2^)	− 0.16 (0.54)	− 1.27–0.95	0.77	R^2^ ≈ 0.01	1.08
		Total energy (J)	0.000098 (0.0018)	− 0.0036–0.0038	0.96	R^2^ ≈ 0.00	1.30
	Multivariate	Protocol (high vs. low)	− 16.7 (2.85)	− 22.6 to –10.9	< 0.001	Adj. R^2^ = 0.63*	1.50
		Tumor size (cm^2^)	0.64 (0.39)	− 0.12–1.40	0.10		1.27
		MELD score	− 0.07 (0.37)	− 0.83–0.69	0.85		1.74
		BMI (kg/m^2^)	− 0.16 (0.54)	− 1.27–0.95	0.77		1.08
		Total energy (J)	0.000098 (0.0018)	− 0.0036–0.0038	0.96		1.30
Metastases (n = 32)	Univariate	Protocol (high vs. low)	− 13.6 (4.42)	− 21.3 to –6.2	0.004	R^2^ = 0.51	1.00
		Tumor size (cm^2^)	+ 0.98 (0.56)	− 0.12–2.08	0.08	R^2^ = 0.09	2.38
		BMI (kg/m^2^)	− 0.21 (0.34)	− 0.90–0.48	0.54	R^2^ = 0.002	2.36
		Total energy (J)	− 0.000021 (0.000068)	− 0.00016–0.00012	0.76	R^2^ ≈ 0.00	1.46
	Multivariate	Protocol (high vs. low)	− 12.8 (4.89)	− 22.4 to − 3.2	0.007	Adj. R^2^ = 0.56*	1.12
		Tumor size (cm^2^)	0.81 (0.61)	− 0.40–2.02	0.18		2.38
		BMI (kg/m^2^)	− 0.17 (0.32)	− 0.84–0.50	0.62		2.36
		Total energy (J)	− 0.000019 (0.000066)	− 0.00015–0.00011	0.78		1.46

β, regression coefficient; SE, standard error; CI, confidence interval; R^2^, coefficient of determination; Adj. R^2^, adjusted coefficient of determination; VIFs; BMI, body mass index; MELD, Model for End-Stage Liver Disease. *Indicates that the reported value refers to the adjusted R^2^ for the multivariate model (overall model fit)

### Exploratory survival analysis

In exploratory analyses over 6 months, the high-power protocol showed numerically lower hazards (HCC hazard ratio (HR) = 0.64, 95% CI: 0.38–1.12; metastases HR = 0.72, 95% CI: 0.38–1.31), without statistical significance. These exploratory analyses were retained in the main manuscript for transparency but are underpowered; no survival inference can be drawn.

## Discussion

### Principal findings and interpretation

This study evaluated the impact of high-power MWA on ablation zone stability in patients with HCC or liver metastases. Follow-up imaging showed that high-power led to reduced contraction of ablation zones, consistent across cohorts and significant after adjustment. This indicates that high-power MWA yielded more stable ablation zones, supporting that greater thermal output enhances coagulation and limits shrinkage. These findings align with prior work showing that high-power MWA produces larger and more homogenous ablation zones and show  reduced heat-sink sensitivity [4, 5, 10]. Whereas earlier studies primarily focused on size, geometry and predictability of ablation zone, our results indicate that the same principles extend to temporal stability of the ablation zone.

### Energy metrics and technical implications

A potential concern was misclassification within the transitional 80–100 W range; however, both protocols followed predefined gradual escalation strategies with distinct upper limits: low-power ablations were initiated at 60 W and never exceeded 100 W, whereas high-power procedures were initiated at 80 W and were consistently escalated to at least 120 W. This escalation pattern resulted in a clear separation of achieved power density between protocols, as reflected in the energy metrics. This is reflected by significant differences in applied wattage and power density (Fig. 2 and Table 2), supporting protocol-based rather than arbitrary categorization. This approach is consistent with the reported heterogeneity of clinical MWA power settings across institutions [16]. Although total energy and energy/cm^2^ overlapped, power density (W/cm^2^) was significantly higher in high-power HCC ablations (*p* < 0.001, RBC = 0.87, 95% CI: 0.71–0.94) and trended higher in metastases (*p* = 0.089, RBC = 0.41, 95% CI: − 0.03–0.71). This concept is supported by technical ablation studies demonstrating that high-power (> 100 W) MWA produces larger and more uniform ablation zones compared with lowpower protocols, as shown in recent clinical experience with 150 W MWA systems [17]. Ablation times were comparable, indicating that higher wattage achieved greater coagulation without prolonging procedures. High-power MWA may also reduce variability related to tissue heterogeneity and local perfusion, which are recognized as important technical factors influencing ablation extent and procedural outcome in consensus and standards documents on image-guided thermal ablation [8]. By delivering higher instantaneous energy, high-power protocols may achieve more uniform thermal profiles within shorter application times, consistent with experimental in vivo data demonstrating that power–time combinations significantly influence ablation zone size and geometry [18]. While previous investigations primarily focused on ablation zone size  and geometry, the present findings suggest that similar principles apply to the temporal stability of the ablation zone, an aspect that has received comparatively limited attention.

### Predictors of ablation zone stability

In the HCC cohort treated with high-power MWA, total energy delivery correlated moderately and negatively with contraction (Spearman’s ρ = − 0.48; *p* = 0.03), supporting the link between higher energy and greater stability. Prior studies likewise reported energy–size relationships modulated by cirrhosis and other tissue characteristics [19]. Multivariable regression confirmed high-power MWA as an independent predictor after adjustment for BMI, tumor size, MELD, and energy. Tumor size was significant in univariable HCC analysis but lost significance after adjustment, suggesting mitigation of size-related effects under high-power protocols. MELD and BMI were not predictive, indicating that contraction dynamics are primarily technical rather than patient-driven. Total energy was also non-predictive once power density was considered, highlighting the role of instantaneous delivery.

Exploratory survival analyses were limited by few events and short follow-up. No conclusions can therefore be drawn, and larger cohorts with longer follow-up are required.

### Clinical relevance and integration with prior work

A recent systematic review emphasized that margins ≥ 5 mm reduce progression in both HCC and colorectal liver metastases [20]. In unresectable HCC beyond the Milan criteria, TACE plus MWA significantly improved overall and progression-free survival compared with TACE alone [9], with recent meta-analytic evidence further supporting a synergistic therapeutic effect of combined TACE and MWA in unresectable HCC [7]. For colorectal liver metastases, Vogl et al. [21] showed that preablation TACE combined with MWA outperforms MWA alone; in unresectable disease, it prolonged hepatic progression-free survival (13.8 vs. 8.1 months, *p* = 0.03) with a favorable trend toward longer overall survival (30 vs. 26 months). Our data add that higher ablation power further improves ablation zone stability across tumor types. Given evidence that wider margins reduce recurrence [20], ablation zone stability may represent a reproducible radiologic marker of local treatment effect; however, its relationship to recurrence requires prospective validation. The observation that higher wattages did not lengthen procedures highlights a practical advantage, enabling greater stability without additional procedural burden.

### Strengths and limitations

This retrospective, single-center study with a modest cohort limits generalizability. Requiring complete MRI follow-up likely favored more adherent patients and may have introduced selection bias toward favorable outcomes. Although nominal wattage overlapped at 80–100 W, all low-power cases were limited to 100 W or less and all high-power cases escalated to at least 120 W. Delivered parameters confirmed clear separation between protocols (Fig. 2, Table 2). No dedicated sensitivity analysis excluded the 80–100 W subgroup, but consistent differences across applied parameters support the validity of the stratification.

Volumetric segmentation was not performed. Ablation size was therefore assessed using a two-dimensional elliptical method, which is widely applied in retrospective MWA cohorts [5, 19]. Early MWA series relied on two-dimensional diameter measurements, and systematic reviews confirm that diameter- and margin-based approaches remain common in clinical ablation studies [5, 20]. In line with consensus recommendations, standardized terminology and reporting were applied while acknowledging the frequent use of two-dimensional methods in retrospective analyses [15]. Our approach is therefore consistent with prior literature, although reproducibility testing and volumetric analysis should be pursued prospectively.

All ablations were performed by one experienced interventional radiologist, ensuring consistency but limiting external generalizability. Multiple probe types and system generations may have introduced heterogeneity. Survival analyses were exploratory and underpowered, restricted to six months, which precludes long-term inference. MRI was acquired at standardized intervals (T1, T2, T3). Only the T1–T3 change defined the primary endpoint, whereas T2 served as a routine control. Median (IQR) values were used to describe the TACE–MWA interval due to skewed distributions. Adoption of high-power protocols later in the study period raises possible temporal confounding, although consistent effects suggest robustness. Interobserver reliability was not assessed in this study. Ablation zone measurements were performed by a single experienced reader blinded to clinical data and treatment protocol, using standardized MRI time points and a consistent measurement approach across all cases. While this ensured internal consistency and feasibility within the retrospective study design, it precludes formal quantification of measurement reproducibility (e.g., intraclass correlation coefficient or Cohen’s kappa) and may limit generalizability. Future prospective studies should incorporate inter- and intra-observer agreement analyses, ideally supported by volumetric segmentation on isotropic imaging datasets.

Strengths include standardized MRI protocols, blinded assessment, and robust bootstrapping. Few prior studies have systematically examined ablation zone stability, and our findings suggest it may serve as a complementary exploratory imaging endpoint of the short- to mid-term treatment effect. However, ablation zone stability should be regarded as an exploratory imaging marker, and prospective validation is required before clinical implementation.

### Future directions

Future studies should validate ablation zone stability in larger prospective multicenter cohorts with longer follow-up. Thin-slice isotropic MRI would allow volumetric segmentation and reproducibility testing, while inter- and intra-observer analyses could confirm reliability. Correlating stability with oncologic outcomes such as Modified Response Evaluation Criteria in Solid Tumors-defined progression, margin adequacy, and progression-free survival, as well as apparent diffusion coefficient values, may clarify its role as a surrogate endpoint. This distinction is essential to avoid overinterpretation of imaging-based findings and underscores the need for prospective validation with oncologic endpoints.

## Conclusion

High-power MWA after preablation TACE was associated with significantly greater ablation zone stability in both primary and metastatic liver tumors. These findings suggest that higher power is associated with reduced post-procedural contraction. Ablation zone contraction emerges as an exploratory imaging marker that warrants validation in prospective studies with longer follow-up and correlation to established oncologic outcomes.

## Data Availability

The data presented in this study are available from the corresponding author upon reasonable request.

## Abbreviations

BMI: Body mass index
CI: Confidence interval
CT: Computed tomography
HCC: Hepatocellular carcinoma
HR: Hazard ratio
IQR: Interquartile range
MELD: Model for End-Stage Liver Disease
MRI: Magnetic resonance imaging
MWA: Microwave ablation
R^2^: Coefficient of determination
RBC: Rank-biserial correlation
RFA: Radiofrequency ablation
SD: Standard deviation
SE: Standard error
TACE: Transarterial chemoembolization
β: Regression coefficient
x^2^: Chi-squared test
φ: Phi coefficient
